# Efficacy and Toxicity of Three Induction Chemotherapy Regimens in Locoregionally Advanced Nasopharyngeal Carcinoma: Outcomes of 10-Year Follow-Up

**DOI:** 10.3389/fonc.2021.765378

**Published:** 2021-10-14

**Authors:** Hao Peng, Binbin Chen, Shuiqing He, Li Tian, Ying Huang

**Affiliations:** ^1^ Center for Translational Medicine, Precision Medicine Institute, The First Affiliated Hospital, Sun Yat-sen University, Guangzhou, China; ^2^ Department of Clinical Nutrition, Sun Yat-sen University Cancer Center, State Key Laboratory of Oncology in South China, Collaborative Innovation Center of Cancer Medicine, Guangzhou, China; ^3^ Department of Radiation Oncology, Sun Yat-sen University Cancer Center, State Key Laboratory of Oncology in South China, Collaborative Innovation Center of Cancer Medicine, Guangzhou, China; ^4^ Imaging Diagnosis and Interventional Center, Sun Yat-sen University Cancer Center, State Key Laboratory of Oncology in South China, Collaborative Innovation Center of Cancer Medicine, Guangzhou, China

**Keywords:** nasopharyngeal carcinoma, induction chemotherapy, 10-year outcomes, radiotherapy, concurrent chemoradiotherapy (CCRT)

## Abstract

**Background/Objective:**

We aimed to compare the 10-year survival outcomes of induction docetaxel plus cisplatin and 5-fluorouracil (TPF), docetaxel plus cisplatin (TP), and cisplatin plus 5-fluorouracil (PF) regimens additional to concurrent chemoradiotherapy (CRT) in locoregionally advanced nasopharyngeal carcinoma (NPC).

**Methods:**

Eligible patients with newly diagnosed stage III-IVA NPC were included. Propensity score matching (PSM) was used to balance prognostic covariates. Survival outcomes and toxicities between different groups were compared.

**Results:**

A total of 855 patients between 2009 and 2012 were included, with 395 (46.2%), 258 (30.2%), and 202 (23.6%) receiving TPF plus CRT, TP plus CRT, and PF plus CRT regimens, respectively. After a median follow-up of 111.8 months, multivariate analysis both in the whole cohort and PSM selected 202 pairs showed that TPF plus CRT and TP plus CRT achieved significantly better 10-year overall survival (OS) than PF plus CRT. Sensitivity analysis after excluding patients with T3-4N0 disease demonstrated that TPF plus CRT still achieved significantly better OS than PF plus CRT (HR, 0.580; 95% CI, 0.395-0.852; *P* = 0.005), while the difference between TP plus CRT and PF plus CRT was marginally significant (HR, 0.712; 95% CI, 0.503-1.008; *P* = 0.056). With regard to toxicity profile, PF regimen achieved the lowest grade 3–5 toxicities (27.3%).

**Conclusion:**

TPF plus CRT and TP plus CRT were better than PF plus CRT in improving the 10-year OS of patients with stage III-IVA NPC.

## Background

As an aggressive and relatively rare head and neck cancer, nasopharyngeal carcinoma (NPC) has an extremely unbalanced geographical distribution worldwide; it is endemic in Southern China and Southeast Asia but very rare in western countries (1, 2). Different from other head and neck cancers, nondisseminated NPC is cured by radiotherapy. A 10-year overall survival (OS) of 87.1–100% could be achieved in patients with stage I-II disease; however, the corresponding survival outcome of patients with stage III-IVA disease was only 75.5–55.6%, and distant metastasis has come as the main failure patter (3). Unfortunately, more than 80% of patients presented with advanced diseases at initial diagnosis (2, 4). Therefore, how to reduce distant metastasis and improve the therapeutic outcomes of patients with advanced disease have been widely studied.

Induction chemotherapy (IC), usually a combination of two or three cytotoxic drugs, is given before radiotherapy to eliminate clinically undetectable micrometastatic lesions, thereby reducing the rate of distant failure and improving survival. Indeed, several phase III clinical trials conducted in recent years have showed that IC additional to concurrent chemoradiotherapy (CRT) could improve both distant metastasis-free survival (DMFS) and OS (5–9). This evidence strengthened the role of IC in locoregionally advanced NPC, and IC plus CRT was therefore approved as the preferable treatment strategy worldwide for advanced NPC. Despite these advances, the most effective IC regimen, however, is still unknown since various regimens all achieved positive results (5–8, 10, 11). A previously retrospective study uncovered that a triple combination of docetaxel plus cisplatin and 5-fluorouracil (TPF) was better than docetaxel plus cisplatin (TP) and cisplatin plus 5-fluorouracil (PF) regimens in patients with stage III-IVA NPC (excluding T3N0) (12). However, the insufficient follow-up duration (median, 46.1 months) and inclusion of patients who did not receive concurrent chemotherapy made these results inconclusive.

Based on this premise, we conducted this study to compare the 10-year survival outcomes of patients with locoregionally advanced NPC receiving induction TPF, TP, or PF plus CRT in the era of intensity-modulated radiotherapy (IMRT).

## Results

### Baseline Information of Included Patients

A total of 855 patients treated between April 2009 and December 2012 were included in our study, with 395 (46.2%) receiving TPF plus CRT, 258 (30.2%) receiving TP plus CRT, and 202 (23.6%) receiving PF plus CRT. Baseline information of these patients is shown in **Table 1**. The whole cohort had a median age of 44 years and a male-to-female ratio of 3.3. The PF plus CRT group had significantly lower percentages of T4 (29.4% *vs*. 39.3% *vs*. 35.2%, *P* = 0.007), N3 (14.7% *vs*. 20.7% *vs*. 23.3%, *P* = 0.173), and stage IVA (41.1% *vs*. 55.7% *vs*. 52.0%, *P* = 0.001) diseases compared with TPF plus CRT and TP plus CRT groups. More patients in the TPF plus CRT group received three or more cycles than in the TP plus CRT and PF plus CRT groups (69.6% *vs*. 22.3% *vs*. 26.7%, *P* < 0.0001). Notably, the TP plus CRT group had the lowest percentage of patients receiving a cumulative cisplatin dose (CCD) ≥ 200 mg/m^2^ (8.9% *vs*. 23.0% *vs*. 21.7%, *P* < 0.0001). A total of 278 patients received weekly cisplatin/nedaplatin during radiotherapy, with 80 (20.3%), 136 (52.7%), and 62 (29.7%) in the TPF, TP, and PF groups, respectively.

**Table 1:** Baseline information of 855 patients receiving different IC regimens.

Characteristics	TPF	PF	TP	*P* value
	No. (%)	No. (%)	No. (%)	
Gender				0.589
Male	304 (77.0)	150 (74.3)	202 (78.3)	
Female	91 (23.0)	52 (25.7)	56 (21.7)	
Age (years)				0.461^b^
Median (range)	43 (16-72)	46 (12-72)	45 (18-76)	
≥ 50	114 (28.9)	71 (35.1)	77 (29.8)	
< 50	281 (71.1)	131 (64.9)	181 (70.2)	
Smoking				0.584
Yes	158 (40)	72 (35.6)	100 (38.8)	
No	237 (60)	130 (64.4)	158 (61.2)	
Alcohol intake				0.781
Yes	54 (13.7)	24 (11.9)	36 (14.0)	
No	341 (86.3)	178 (88.1)	222 (86.0)	
Family history of cancer				0.64
Yes	106 (26.8)	47 (23.3)	66 (25.6)	
No	289 (73.2)	155 (76.7)	192 (74.4)	
T category^a^				0.007
T1	21 (5.3)	13 (6.4)	4 (1.6)	
T2	30 (7.6)	18 (8.9)	20 (7.8)	
T3	189 (47.8)	100 (49.5)	158 (61.2)	
T4	155 (39.3)	71 (35.2)	76 (29.4)	
N category^a^				0.173
N0	18 (4.6)	11 (5.4)	19 (7.4)	
N1	198 (50.1)	91 (45.1)	139 (53.9)	
N2	97 (24.6)	53 (26.2)	62 (24.0)	
N3	82 (20.7)	47 (23.3)	38 (14.7)	
Overall stage^a^				0.001
III	175 (44.3)	97 (48.0)	152 (58.9)	
IVA	220 (55.7)	105 (52.0)	106 (41.1)	
IC cycle				<0.0001
2	120 (30.4)	157 (77.7)	189 (73.3)	
3	243 (61.5)	32 (15.9)	55 (21.3)	
4	32 (8.1)	13 (6.4)	14 (5.4)	
CCD (mg/m^2^)				<0.0001^b^
Median (range)	160 (40-300)	160 (30-300)	160 (30-300)	
≥ 200	91 (23.0)	18 (8.9)	56 (21.7)	
< 200	304 (77.0)	184 (91.1)	202 (78.3)	

IC, induction chemotherapy; TPF, docetaxel plus cisplatin and 5-fluorouracil; PF, cisplatin plus 5-fluorouracil; TP, docetaxel plus cisplatin; CCD, cumulative cisplatin dose during radiotherapy.

a According to the eighth edition of the AJCC/UICC manual.

b P values were calculated by one-way ANOVA.

### Treatment Failure Pattern

Up to the last follow-up (August 20, 2021), the median follow-up duration was 111.8 months (range, 4.57–149.63 months) for the whole cohort and 120.9 months (range, 7.43–149.63 months) for those alive. Among the patients survived, 9.6% (56/586) of them were lost to follow-up, and only 5 (0.9%) of the remaining patients were followed for less than 9 years (range, 105.2–107.6 months). A total of 269 (31.6%) deaths were observed, with 124 (31.4%) in the TPF plus CRT group, 78 (38.6%) in the PF plus CRT group, and 67 (26.0%) in the TP plus CRT group. Moreover, 85 (21.5%), 45 (11.4%), and 40 (10.1%) patients in the TPF plus CRT group suffered distant, local, and regional recurrence, respectively. The corresponding numbers were 49 (24.3%), 23 (11.4%), and 20 (9.9%) in the PF plus CRT group, and 45 (17.4%), 20 (7.8%), and 8 (3.1%) in the TP plus CRT group. Intriguingly, 29 (3.4%) patients still survived after disease progression and salvage treatments, with 18 (4.6%) in the TPF plus CRT group, 4 (2.0%) in the PF plus CRT group, and 7 (2.7%) in the TP plus CRT group. Notably, 39 new events, which accounted for 13.1% of all events, occurred after 5 years, with 21 (5.3%) in the TPF plus CRT group, 11 (5.4%) in the PF plus CRT group, and 7 (2.7%) in the TP plus CRT group (**Supplementary Table S1**).

### Survival Outcomes Comparison

The estimated 10-year OS, DFS, DMFS, and LFFS rates were 67.8%, 64.9%, 78.4%, and 83.8% for the whole cohort, respectively. With regard to the three groups, the estimated 10-year survival rates of TPF *vs*. TP *vs*. PF were 67.7% *vs*. 73.5% *vs*. 60.5% (*P*_TPF *vs*. TP_ = 0.153, *P*_TPF *vs*. PF_ = 0.058, *P*_PF *vs*. TP_ = 0.003) for OS, 63.6% *vs*. 71.1% *vs*. 59.2% (*P*_TPF *vs*. TP_ = 0.055, *P*_TPF *vs*. PF_ = 0.262, *P*_PF *vs*. TP_ = 0.007) for DFS, 77.7% *vs*. 82.3% *vs*. 74.5% (*P*_TPF *vs*. TP_ = 0.209, *P*_TPF *vs*. PF_ = 0.39, *P*_PF *vs*. TP_ = 0.063) for DMFS, and 81.2% *vs*. 89.5% *vs*. 81.8% (*P*_TPF *vs*. TP_ = 0.006, *P*_TPF *vs*. PF_ = 0.968, *P*_PF *vs*. TP_ = 0.015; **Supplementary Figure S1**) for LFFS. After adjusting for various factors by an adjusted Cox proportional hazards model, TPF plus CRT (OS: HR, 0.672; 95% CI, 0.491–0.920; *P* = 0.013; DFS: HR, 0.753; 95% CI, 0.544–0.994; *P* = 0.045) and TP plus CRT (OS: HR, 0.664; 95% CI, 0.478–0.922; *P* = 0.015; DFS: HR, 0.701; 95% CI, 0.510–0.963; *P* = 0.029) were associated with significantly better OS and DFS compared with PF plus CRT (**Table 2**).

**Table 2:** Results of multivariate analysis.

Endpoint	Factor	Hazard ratio (95% CI)	*P* value
OS	Alcohol intake (Yes *vs*. No)	1.421 (1.033-1.950)	0.031
	Age (≥ 50y *vs*. < 50y)	1.351 (1.054-1.730)	0.017
	N category (N2-3 *vs*. N0-1)	1.771 (1.363-2.301)	<0.0001
	Overall stage (IVA *vs*. III)	1.757 (1.354-2.282)	<0.0001
	Treatment (TPF plus CRT *vs*. PF plus CRT)	0.672 (0.491-0.920)	0.013
	Treatment (TP plus CRT *vs*. PF plus CRT)	0.664 (0.478-0.922)	0.015
DFS	Alcohol intake (Yes *vs*. No)	1.477 (1.093-1.998)	0.011
	T category (T3-4 *vs*. T1-2)	1.508 (1.043-2.180)	0.029
	N category (N2-3 *vs*. N0-1)	1.925 (1.501-2.467)	<0.0001
	Overall stage (IVA *vs*. III)	1.644 (1.286-2.101)	<0.0001
	Treatment (TPF plus CRT *vs*. PF plus CRT)	0.753 (0.544-0.994)	0.045
	Treatment (TP plus CRT *vs*. PF plus CRT)	0.701 (0.510-0.963)	0.029
DMFS	Gender (Female *vs*. male)	0.620 (0.416-0.923)	0.018
	N category (N2-3 *vs*. N0-1)	2.458 (1.179-3.364)	<0.0001
	Overall stage (IVA *vs*. III)	1.902 (1.387-2.610)	<0.0001
	Treatment (TPF plus CRT *vs*. PF plus CRT)	0.784 (0.531-1.158)	0.221
	Treatment (TP plus CRT *vs*. PF plus CRT)	0.774 (0.515-1.164)	0.219
LFFS	T category (T3-4 *vs*. T1-2)	2.069 (1.081-3.961)	0.028
	N category (N2-3 *vs*. N0-1)	1.627 (1.120-2.363)	0.011
	Treatment (TPF plus CRT *vs*. PF plus CRT)	1.002 (0.664-1.514)	0.991
	Treatment (TP plus CRT *vs*. PF plus CRT)	0.565 (0.336-0.949)	0.031

OS, overall survival; DFS, disease-free survival; DMFS, distant metastasis-free survival; LFFS, locoregional failure-free survival; CI, confidence interval; IC, induction chemotherapy; CRT, concurrent chemoradiotherapy; Pre-DNA, pre-treatment plasma EBV DNA.

P-values were calculated using an adjusted Cox proportional hazards model with backward elimination, and the following variables were included: gender (female vs. male), age (≥ 50 years vs. < 50 years), smoking (yes vs. no), drinking (yes vs. no), family history of cancer (yes vs. no), T category (T3-4 vs. T1-2), N category (N2-3 vs. N0-1), overall stage (IVA vs. III), cumulative cisplatin dose during radiotherapy (≥ 200 vs. < 200 mg/m^2^), induction chemotherapy cycle (2 vs. 3-4), and treatment groups (TPF plus CRT vs. PF plus CRT, TP plus CRT vs. PF plus CRT).

**Figure 1:**
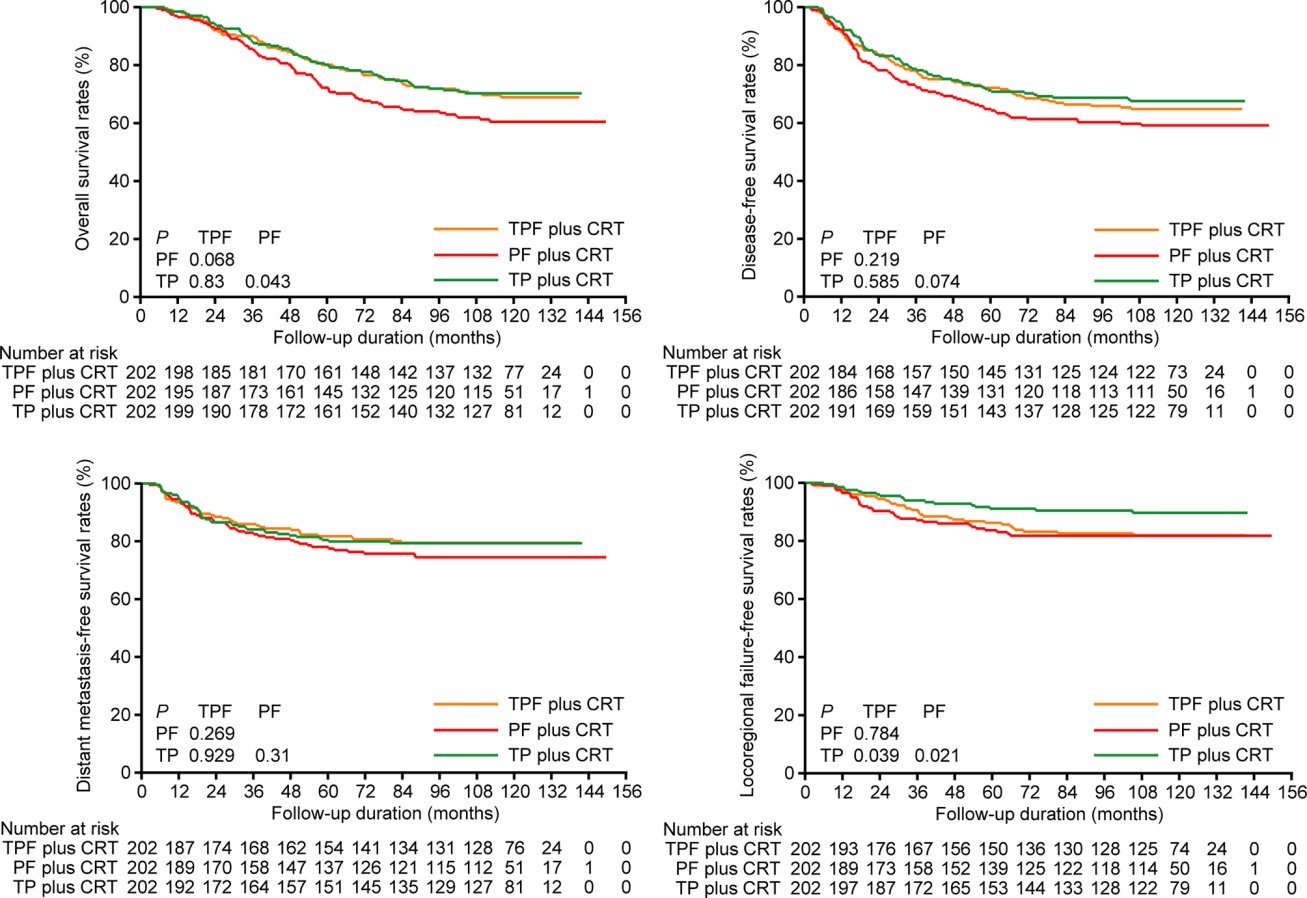
Kaplan-Meier overall survival, disease-free survival, distant metastasis-free survival, and locoregional failure-free survival curves of patients receiving induction TPF, PF, and TP plus CRT in the 202 pairs selected by propensity score matching. TPF, docetaxel plus cisplatin and fluorouracil; PF, cisplatin plus fluorouracil; TP, docetaxel plus cisplatin.

We used PSM to balance independent prognostic factors identified above (tumor stage, alcohol intake, age, and gender) and further performed survival analysis in the selected 202 pairs (**Supplementary Table S2**). Correspondingly, the 10-year OS, DFS, DMFS, and LFFS rates for TPF *vs*. PF *vs*. TP were 68.9% *vs*. 70.3% *vs*. 60.5% (*P*_TPF *vs*. TP_ = 0.83, *P*_TPF *vs*. PF_ = 0.068, *P*_PF *vs*. TP_ = 0.043), 64.8% *vs*. 67.6% *vs*. 59.2% (*P*_TPF *vs*. TP_ = 0.585, *P*_TPF *vs*. PF_ = 0.219, *P*_PF *vs*. TP_ = 0.074), 79.5% *vs*. 79.3% *vs*. 74.5% (*P*_TPF *vs*. TP_ = 0.929, *P*_TPF *vs*. PF_ = 0.269, *P*_PF *vs*. TP_ = 0.310), and 81.9% *vs*. 89.7% *vs*. 81.8% (*P*_TPF *vs*. TP_ = 0.039, *P*_TPF *vs*. PF_ = 0.784, *P*_PF *vs*. TP_ = 0.021; **Figure 1**). Results of multivariate analysis revealed that TPF plus CRT (HR, 0.617; 95% CI, 0.426–0.894; *P* = 0.011) and TP plus CRT (HR, 0.699; 95% CI, 0.498–0.982; *P* = 0.039) groups were associated with significantly improved OS but marginally significant DFS (TPF plus CRT: HR, 0.701; 95% CI, 0.491–1.002; *P* = 0.051; TP plus CRT: HR, 0.738; 95% CI, 0.532–1.025; *P* = 0.07) compared with PF plus CRT (**Supplementary Table S3**).

### Sensitivity Analysis

**Figure 2:**
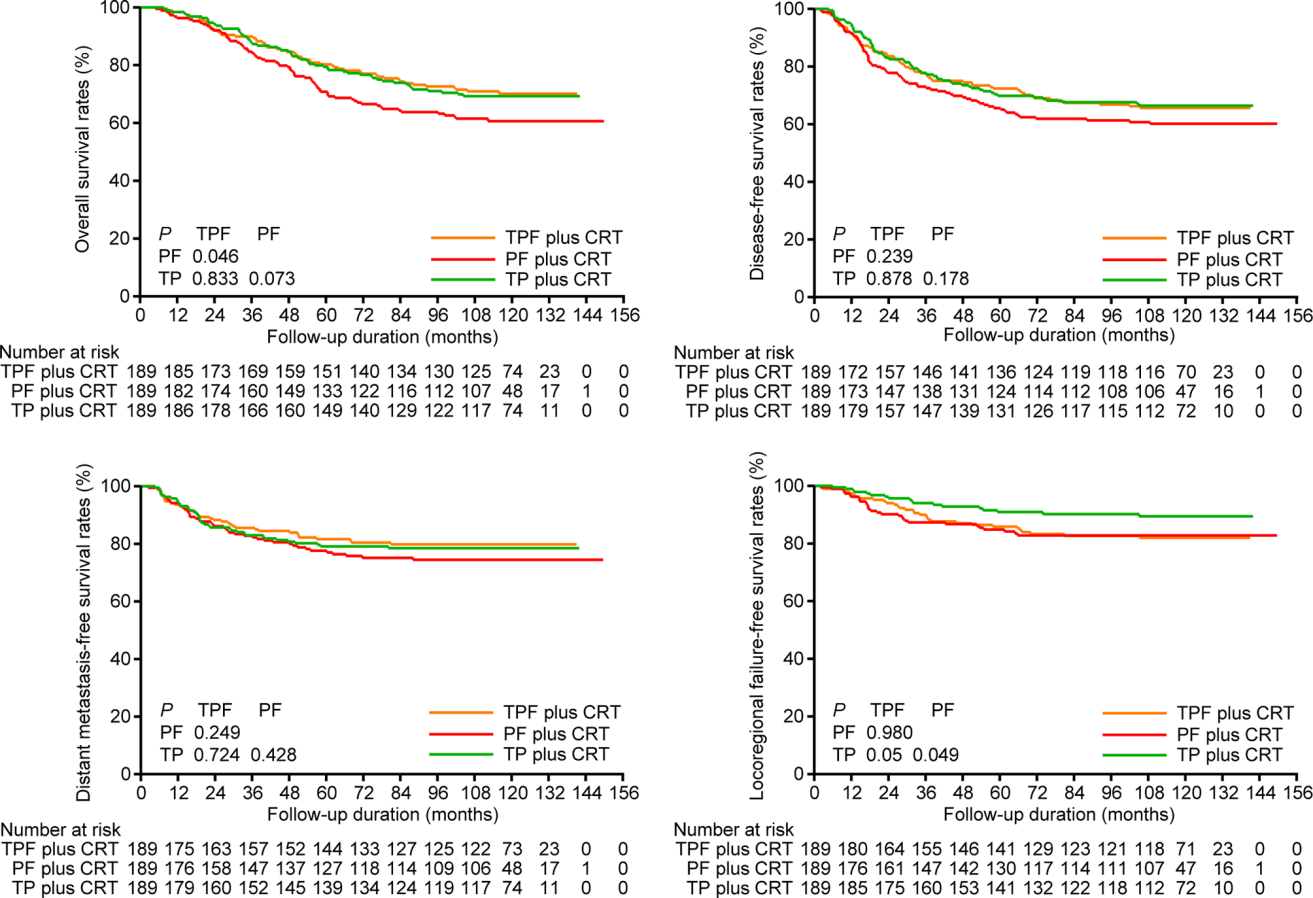
Kaplan-Meier overall survival, disease-free survival, distant metastasis-free survival, and locoregional failure-free survival curves of patients receiving induction TPF, PF, and TP plus CRT in the 189 pairs of sensitivity analysis. TPF, docetaxel plus cisplatin and fluorouracil; PF, cisplatin plus fluorouracil; TP, docetaxel plus cisplatin.

We performed sensitivity analysis by excluding stage T3-4N0 disease, which was regarded as low risk of distant metastasis by previous trials (5, 7). In total, 32 patients were excluded, and 189 pairs were selected by PSM from the remaining patients (**Supplementary Table S4** **)**. Consistent with the results above, TPF plus CRT and TP plus CRT still achieved higher 10-year OS (70.1% *vs*. 69.3% *vs*. 60.6%), DFS (65.7% *vs*. 66.4% *vs*. 60.2%) and DMFS (79.8% *vs*. 78.5% *vs*. 74.4%) rates than PF plus CRT (**Figure 2**). Multivariate analysis demonstrated a significant difference in OS between TPF plus CRT and PF plus CRT (HR, 0.580; 95% CI, 0.395–0.852; *P* = 0.005), while this difference between TP plus CRT and PF plus CRT was marginally significant (HR, 0.712; 95% CI, 0.503–1.008; *P* = 0.056; **Supplementary Table S5**).

### Toxicity Comparison

Treatment adverse events of different IC regimens are shown in **Table 3**. As expected, the PF regimen achieved the lowest percentages of grade 3–5 toxicities (27.3%), and the TP regimen had the highest rate of grade 3–5 toxicities, which were mainly grade 3–5 neutropenia (97.1%) and febrile neutropenia (11.8%). This should be due to the application of a higher dose of docetaxel (75 mg/m^2^). Otherwise, grade 3–5 nonhematologic toxicities were uncommon in the TP group. Compared with the PF regimen, the TPF regimen had higher grade 3–5 neutropenia (35.6% *vs*. 14.7%, *P* < 0.001), leukopenia (27.2% *vs*. 5%, *P* < 0.001), and mucositis (6.3% *vs*. 1.3%, *P* = 0.004). Undoubtedly, docetaxel additional to PF would result in greater toxicities.

**Table 3:** Acute toxicity comparison during induction chemotherapy.

	TPF (n = 239)	TP (n = 34)	PF (n = 238)	*P* value
Overall Grade 3-5 (%)	43.1 (103/239)	/	27.3 (65/238)	< 0.001
Grade 3-5 Hematologic				
Neutropenia	35.6 (85/239)	97.1 (33/34)	14.7 (35/238)	< 0.001
Febrile Neutropenia	1.7 (4/239)	11.8 (4/34)	/	< 0.001
Neutropenia infection	0.8 (2/239)	/	/	/
Leukopenia	27.2 (65/239)	/	5 (12/238)	< 0.001
Anemia	0.4 (1/239)	/	0.4 (1/238)	NS
Thrombocytopenia	0	/	0	NS
Grade 3-5 Non-hematologic				
Diarrhea	7.9 (19/239)	/	/	/
Mucositis	6.3 (15/239)	/	1.3 (3/238)	0.004
Nausea/vomiting	7.5 (18/239)	8.8 (3/34)	4.2 (10/238)	NS
Hepatoxicity	2.5 (6/239)	/	0.8 (2/238)	NS
Allergic reaction	0.8 (2/239)	/	0.4 (1/238)	NS
Fatigue	/	5.9 (2/34)	/	/
Ototoxicity	0	/	0	NS
Nephrotoxicity	0	/	0	NS

TPF, docetaxel plus cisplatin with 5-fluorouracil; TP, docetaxel plus cisplatin; PF, 5-fluorouracil plus cisplatin; NS, not significant.

## Discussion

Our current study reported the 10-year survival outcomes of patients with stage III-IVA NPC receiving different IC regimens plus CRT in the era of IMRT. We found that TPF plus CRT and TP plus CRT achieved significantly better OS than PF plus CRT both in the whole cohort and the selected pairs by PSM. Toxicity analysis showed that the PF regimen had the lowest percentages of grade 3–5 adverse events. To date, our study is the first one to report the 10-year therapeutic outcomes of locoregionally advanced NPC treated by IC plus CRT in the era of IMRT.

Our study only recruited patients receiving induction TPF, TP, and PF regimens because these three regimens have been used most frequently and for the longest time in our center. Their efficacy in locoregionally advanced NPC has also been verified by clinical trials (5, 6, 8–11). Although gemcitabine plus cisplatin (GP) is also effective and may have fewer adverse events (7), the insufficient follow-up duration of patients receiving this regimen precludes them from being enrolled into this study. As previous study showed that two cycles of IC could achieve comparable outcomes as three or more cycles (13), we therefore only recruited patients receiving at least two cycles to reduce the impact of the IC cycle. Consistent with previous findings (13), results of multivariate analysis in our study also did not identify the IC cycle (2 *vs*. 3-4) as an independent prognostic factor. Another interesting finding was that patients receiving the TP regimen achieved lower CCD dose than those receiving the TPF regimen. The mainly responsible reason may be that a higher dose of docetaxel (75 mg/m^2^) may reduce patients’ tolerance to concurrent cisplatin/nedaplatin.

Notably, 39 new events occurred 5 years after radiotherapy, accounting for 13.1% of all events. Therefore, intensive follow-up is still needed after 5 years. Among the 39 new events, distant metastasis only accounted for 30.7% and noncancer-related death accounted for 33.3%. These results indicated that distant metastasis was no longer the main cause of treatment failure after 5 years for patients receiving IC plus CRT, and we should pay attention to noncancer-related death, which may be due to treatment-related sequelae.

When analyzing all the 855 patients together, both the TPF plus CRT and TP plus CRT groups achieved significantly better OS and DFS than the PF plus CRT group. However, some comparisons in PSM or sensitivity analysis only showed marginally significant difference. The main reason contributing to this should be the decreased sample sizes, which reduced statistical power in PSM and sensitivity analysis. Generally, induction TPF and TP regimens should be more effective than the PF regimen, which was consistent with the findings of a meta-analysis that taxanes-based IC could decrease the risk of distant metastasis by above 10% for patients with stage IVA disease (14). Moreover, the effect of docetaxel additional to induction PF regimen has also been verified in head and neck cancers (15–18). Different from previous results that TPF was significantly better than TP with regard to DFS and OS endpoints (12), survival endpoints except LFFS did not significantly differ between the TPF plus CRT and TP plus CRT groups. There may be three reasons attributing to this discrepancy. First, the follow-up duration was much longer in our study. Second, patients with stage T3N0 were excluded in that study. Third, patients who did not receive concurrent chemotherapy were also included in that study.

Limitations of our study should also be addressed. First, many potential bias may exist in our retrospective study. We therefore set strict enrollment criteria and balanced various prognostic factors by PSM to reduce bias. Moreover, we performed sensitivity analysis to further validate our findings. Second, toxic data during IC are unavailable for most of the patients due to the retrospective nature of our study. We therefore extracted these data from previously published clinical trials. To minimize the impact of ethnic differences on adverse events, we only recruited the three clinical trials (5, 6, 10) conducted in the endemic area (mainly South China). Despite this, this result should be interpreted discreetly. Third, the relatively small sample size, especially in PSM and sensitivity analysis, precluded the production of significant differences for some endpoints, although the survival curves showed obvious differences.

## Materials and Methods

### Patient Inclusion Criteria

We retrospectively reviewed the data of patients with newly diagnosed NPC who were treated at our center between 2009 and 2012. Patients would be enrolled for this study after meeting the following criteria: (1) newly diagnosed stage III-IVA disease; (2) receiving IC plus CRT; (3) IC regimens were TPF, TP, and PF and IC cycles ≥ 2; (4) concurrent chemotherapy regimen should be single-agent platinum; (5) treated by IMRT and received a total dose of at least 66 Gy; (6) no other malignancy. Notably, we included patients receiving TPF, TP, and PF regimens because they were most frequently used at that time. Moreover, the efficacy of these regimens has also been validated in randomized clinical trials. The protocol of our study was approved by the Research Ethics Committee of our center, and all the analyses were carried out in accordance with the Declaration of Helsinki. Written informed consent was obtained from all patients.

### Pretreatment Staging Workup

Potential patients with indicated symptoms in our hospital would receive routine staging workup including physical examination, enhanced magnetic resonance imaging (MRI) of head and neck, chest computed tomography (CT) or X-ray, abdominal CT or sonography, and whole body bone scan. ^18^F-FDG positron emission tomography (PET)-CT would also be recommended to patients who presented with massive lymph node or bilateral cervical lymph node metastasis. Patients were restaged by one radiologist (LT) and one radiation oncologist (YH) separately, both with more than 10-year experience in the diagnosis and treatment of NPC at our center, based on the imaging data and the eighth edition of the International Union against Cancer/American Joint Committee on Cancer (UICC/AJCC) manual (19).

### Induction and Concurrent Chemotherapy Treatment

IC was delivered every 3 weeks for two to four cycles, and the regimens consisted of docetaxel (75 mg/m^2^, d1) plus cisplatin (75 mg/m^2^, d1), cisplatin (80 mg/m^2^ d1) plus 5-fluorouracil (1,000 mg/m^2^ d1-d5, 120 h infusion), or docetaxel (60–75 mg/m^2^, d1) plus cisplatin (60–75 mg/m^2^, d1) and 5-fluorouracil (600–750 mg/m^2^, d1-d5, 120-h infusion). Concurrent chemotherapy was delivered during radiotherapy and consisted of weekly cisplatin/nedaplatin (30–40 mg/m^2^, d1) or tri-weekly cisplatin/nedaplatin (80–100 mg/m^2^, d1).

### Radiotherapy

All the patients received pre-radiotherapy evaluation to exclude any contraindication. IMRT was delivered using the simultaneous integrated boost (SIB) technique. The prescribed doses were 66–70 Gy at 2.0–2.27 Gy per fraction to the planning target volume (PTV) of nasopharynx lesion and metastatic neck lymph nodes, 56–60 Gy at 30–35 fractions to the PTV of clinically high-risk regions, and 50–56 Gy at 30–35 fractions to the PTV of clinically low-risk regions. Radiotherapy fractions were delivered once per day from Monday to Friday each week.

### Toxicity of Induction Chemotherapy

Due to the retrospective nature of our study, IC-related adverse events were not recorded for most of the patients. Therefore, we extracted toxicity data from three previous clinical trials conducted in endemic areas (mainly in South China) (5, 6, 10) to perform indirect comparisons between TPF, TP, and PF regimens.

### Follow-Up Strategy and Endpoints

Patients finishing the treatment would be followed according to the institutional follow-up strategies, which included enhanced MRI of head and neck, chest CT or X-ray, abdominal CT or sonography, and whole bone scan (optional) every 3 months during the first 2 years after radiotherapy, every 6 months during the third to fifth years, and annually thereafter. For patients who lived far away from our hospital, we recommended them to receive these imaging workups at local medical centers and they would be followed by telephone. Disease recurrence including local, regional, and distant metastasis (except bone) was diagnosed by pathology. Bone metastasis was mainly confirmed by imaging methods like MRI, CT, or PET-CT.

Endpoints evaluated at our study included OS (time from diagnosis to death), disease-free survival (DFS, time from diagnosis to disease progression including noncancer-related death), distant metastasis-free survival (DMFS, time from diagnosis to first distant failure), and locoregional failure-free survival (LFFS, time from diagnosis to first local or regional recurrence or both).

### Statistical Methods

T-test or one-way ANOVA was applied to determine the difference between continuous variables, and chi-square test or Fisher’s exact test was used for categorical variables. Propensity score matching (PSM) (20) was employed to balance covariates (T category, N category, overall stage, gender, age, alcohol intake) between the three groups. Estimated 10-year survival outcomes of OS, DFS, DMFS, and LFFS were obtained from Kaplan-Meier methods, and the differences were compared by log-rank test. Independent prognostic factors and their corresponding hazard ratios (HRs) and 95% confidence intervals (CIs) were identified by the multivariate Cox proportional hazard model. All statistical analyses were conducted using the Stata Statistical Package 12 (StataCorp LP, College Station, TX, USA), and a two-sided *P* < 0.05 indicated statistical significance.

## Conclusion

Based on the 10-year follow-up, our current study reported and compared the efficacy of three IC regimens and uncovered that TPF plus CRT and TP plus CRT were better than PF plus CRT in improving the OS of patients with locoregionally advanced NPC. Further comparisons of TPF or TP with the GP regimen by future studies are needed to identify the optimal treatment strategy for NPC patients with locoregionally advanced disease.

## Data Availability Statement

The raw data supporting the conclusions of this article will be made available by the authors, without undue reservation.

## Ethics Statement

The studies involving human participants were reviewed and approved by Sun Yat-sen University Cancer Center. Written informed consent to participate in this study was provided by the participants’ legal guardian/next of kin.

## Author Contributions

HP and YH contributed to study design. BC, SH, and LT collected the study data. HP, BC, and YH contributed to data analysis and interpretation. BC and SH contributed to manuscript writing. HP and YH reviewed the manuscript and contributed to quality control. All authors contributed to the article and approved the submitted version.

## Funding

This work was funded by the National Natural Science Foundation of China (82002981).

## Conflict of Interest

The authors declare that the research was conducted in the absence of any commercial or financial relationships that could be construed as a potential conflict of interest.

## Publisher’s Note

All claims expressed in this article are solely those of the authors and do not necessarily represent those of their affiliated organizations, or those of the publisher, the editors and the reviewers. Any product that may be evaluated in this article, or claim that may be made by its manufacturer, is not guaranteed or endorsed by the publisher.
