# Supplementary material for: Efficacy and Toxicity of Three Induction Chemotherapy Regimens in Locoregionally Advanced Nasopharyngeal Carcinoma: Outcomes of 10-Year Follow-Up
Source: Front Oncol. 2021 Oct 14;11:765378. doi: 10.3389/fonc.2021.765378 (PMC8551638; doi:10.3389/fonc.2021.765378)
Supplement: Supplementary file 1 [file DataSheet_1.docx]

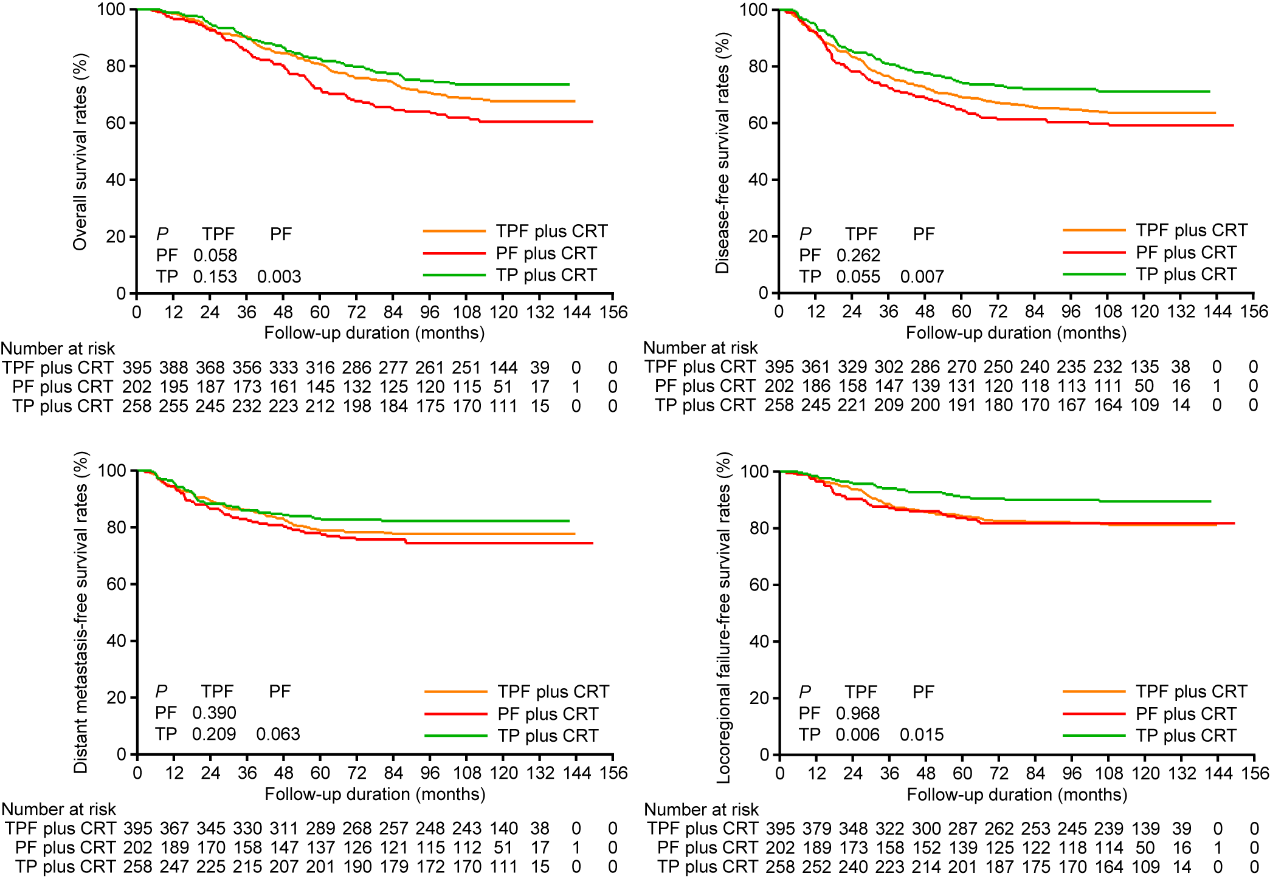


**Supplementary Figure S1**. Kaplan-Meier overall survival, disease-free survival, distant metastasis-free survival and locoregional failure-free survival curves of patients receiving induction TPF, PF and TP plus CRT in the whole cohort. TPF, docetaxel plus cisplatin and fluorouracil; PF, cisplatin plus fluorouracil; TP, docetaxel plus cisplatin.
