# Supplementary material for: Efficacy and Toxicity of Three Induction Chemotherapy Regimens in Locoregionally Advanced Nasopharyngeal Carcinoma: Outcomes of 10-Year Follow-Up
Source: Front Oncol. 2021 Oct 14;11:765378. doi: 10.3389/fonc.2021.765378 (PMC8551638; doi:10.3389/fonc.2021.765378)
Supplement: Supplementary file 2 [file Table_1.docx]

Supplementary Table S1. Failure patterns of 39 new events after 5 years.

| Pattern | TPF plus CRT | PF plus CRT | TP plus CRT |
| --- | --- | --- | --- |
|  | No. (%) | No. (%) | No. (%) |
| Non-cancer death | 9 (42.9) | 2 (18.2) | 2 (28.6) |
| Distant only | 2 (9.5) | 6 (54.5) | 2 (28.6) |
| Local only | 6 (28.6) | 2 (18.2) | 3 (42.8) |
| Local + regional | 2 (9.5) | 1 (9.1) | 0 (0) |
| Distant + regional | 1 (4.8) | 0 (0) | 0 (0) |
| Local + distant | 1 (4.8) | 0 (0) | 0 (0) |

Abbreviations: TPF, docetaxel plus cisplatin and 5-fluorouracil; PF, cisplatin plus 5-fluorouracil; TP, docetaxel plus cisplatin; CRT, concurrent chemoradiotherapy.

Supplementary Table S2. Baseline information of 202 pairs receiving different IC regimens.

| Characteristics | TPF | PF | TP | *P* value |
| --- | --- | --- | --- | --- |
|  | No. (%) | No. (%) | No. (%) |  |
| Gender |  |  |  | 0.347 |
| Male | 161 (79.7) | 150 (74.3) | 160 (79.2) |  |
| Female | 41 (20.3) | 52 (25.7) | 42 (20.8) |  |
| Age (y) |  |  |  | 0.528^b^ |
| Median (range) | 44 (18-72) | 46 (12-72) | 45 (18-76) |  |
| ≥ 50 | 65 (32.2) | 71 (35.1) | 69 (34.2) |  |
| < 50 | 137 (67.8) | 131 (64.9) | 133 (65.8) |  |
| Smoking |  |  |  | 0.359 |
| Yes | 86 (42.6) | 72 (35.6) | 80 (39.6) |  |
| No | 116 (57.4) | 130 (64.4) | 122 (60.4) |  |
| Alcohol intake |  |  |  | 0.744 |
| Yes | 22 (10.9) | 24 (11.9) | 27 (13.4) |  |
| No | 180 (89.1) | 178 (88.1) | 175 (86.6) |  |
| Family history of cancer |  |  |  | 0.838 |
| Yes | 44 (21.8) | 47 (23.3) | 49 (24.3) |  |
| No | 158 (78.2) | 155 (76.7) | 153 (75.7) |  |
| T category^a^ |  |  |  | 0.204 |
| T1 | 11 (5.4) | 13 (6.4) | 2 (1.0) |  |
| T2 | 16 (7.9) | 18 (8.9) | 17 (8.4) |  |
| T3 | 104 (51.5) | 100 (49.5) | 108 (53.5) |  |
| T4 | 71 (35.2) | 71 (35.2) | 75 (37.1) |  |
| N category^a^ |  |  |  | 0.532 |
| N0 | 8 (3.9) | 11 (5.4) | 13 (6.4) |  |
| N1 | 101 (50.0) | 91 (45.1) | 109 (54.0) |  |
| N2 | 49 (24.3) | 53 (26.2) | 43 (21.3) |  |
| N3 | 44 (21.8) | 47 (23.3) | 37 (18.3) |  |
| Overall stage^a^ |  |  |  | 1.0 |
| III | 97 (48.0) | 97 (48.0) | 97 (48.0) |  |
| IVA | 105 (52.0) | 105 (52.0) | 105 (52.0) |  |
| IC cycle |  |  |  | < 0.0001 |
| 2 | 56 (27.7) | 157 (77.7) | 145 (71.8) |  |
| 3 | 127 (62.9) | 32 (15.9) | 45 (22.2) |  |
| 4 | 19 (9.4) | 13 (6.4) | 12 (6.0) |  |
| CCD (mg/m^2^) |  |  |  | < 0.0001^b^ |
| Median (range) | 160 (60-300) | 160 (30-300) | 160 (30-300) |  |
| ≥ 200 | 46 (22.8) | 18 (8.9) | 47 (23.3) |  |
| < 200 | 156 (77.2) | 184 (91.1) | 155 (76.7) |  |

Abbreviations: IC, induction chemotherapy; TPF, docetaxel plus cisplatin and 5-fluorouracil; PF, cisplatin plus 5-fluorouracil; TP, docetaxel plus cisplatin. CCD, cumulative cisplatin dose during radiotherapy.

^a^According to the 8^th^ edition of AJCC/UICC manual.

^b^*P* values were calculated by one-way ANOVA.

Supplementary Table S3. Results of multivariate analysis in the 202 pairs.

| Endpoint | Factor | Hazard ratio (95% CI) | *P* value |
| --- | --- | --- | --- |
| OS | Alcohol intake (Yes vs. No) | 1.667 (1.151-2.412) | 0.007 |
|  | N category (N2-3 vs. N0-1) | 1.670 (1.232-2.263) | 0.001 |
|  | Overall stage (IVA vs. III) | 1.844 (1.351-2.516) | < 0.0001 |
|  | Treatment (TPF plus CRT vs. PF plus CRT) | 0.617 (0.426-0.894) | 0.011 |
|  | Treatment (TP plus CRT vs. PF plus CRT) | 0.699 (0.498-0.982) | 0.039 |
| DFS | Alcohol intake (Yes vs. No) | 1.752(1.230-2.495) | 0.002 |
|  | T category (T3-4 vs. T1-2) | 1.669 (1.068-2.609) | 0.025 |
|  | N category (N2-3 vs. N0-1) | 1.806 (1.350-2.416) | < 0.0001 |
|  | Overall stage (IVA vs. III) | 1.784 (1.331-2.392) | < 0.0001 |
|  | Treatment (TPF plus CRT vs. PF plus CRT) | 0.701 (0.491-1.002) | 0.051 |
|  | Treatment (TP plus CRT vs. PF plus CRT) | 0.738 (0.532-1.025) | 0.07 |
| DMFS | Gender (Female vs. male) | 0.567 (0.349-0.923) | 0.022 |
|  | N category (N2-3 vs. N0-1) | 2.289 (1.587-3.302) | < 0.0001 |
|  | Overall stage (IVA vs. III) | 2.024 (1.387-2.953) | < 0.0001 |
|  | Treatment (PF plus CRT vs. TPF plus CRT) | 0.755 (0.472-1.208) | 0.242 |
|  | Treatment (TP plus CRT vs. TPF plus CRT) | 0.855 (0.560-1.306) | 0.468 |
| LFFS | Overall stage (IVA vs. III) | 2.015 (1.288-3.152) | 0.002 |
|  | Treatment (PF plus CRT vs. TPF plus CRT) | 0.936 (0.579-1.510) | 0.785 |
|  | Treatment (TP plus CRT vs. TPF plus CRT) | 0.524 (0.299-0.918) | 0.024 |

Abbreviations: OS, overall survival; DFS, disease-free survival; DMFS, distant metastasis-free survival; LFFS, locoregional failure-free survival; CI, confidence interval; IC, induction chemotherapy; CRT, concurrent chemoradiotherapy; Pre-DNA, pre-treatment plasma EBV DNA.

*P*-values were calculated using an adjusted Cox proportional hazards model with backward elimination and the following variables were included: gender (female vs. male), age (≥ 50y vs. < 50y), smoking (yes vs. no), drinking (yes vs. no), family history of cancer (yes vs. no), T category (T3-4 vs. T1-2), N category (N2-3 vs. N0-1), overall stage (IVA vs. III), cumulative cisplatin dose during radiotherapy (≥ 200 vs. < 200 mg/m^2^), induction chemotherapy cycle (2 vs. 3-4) and treatment groups (TPF plus CRT vs. PF plus CRT, TP plus CRT vs. PF plus CRT)

Supplementary Table S4. Baseline information of 189 pairs.

| Characteristics | TPF | PF | TP | *P* value |
| --- | --- | --- | --- | --- |
|  | No. (%) | No. (%) | No. (%) |  |
| Gender |  |  |  | 0.347 |
| Male | 150 (79.4) | 139 (73.5) | 150 (79.4) |  |
| Female | 39 (20.6) | 50 (26.5) | 39 (20.6) |  |
| Age (y) |  |  |  | 0.612^b^ |
| Median (range) | 44 (18-72) | 46 (12-72) | 45 (18-76) |  |
| ≥ 50 | 60 (31.7) | 66 (34.9) | 63 (33.3) |  |
| < 50 | 129 (68.3) | 123 (65.1) | 126 (66.7) |  |
| Smoking |  |  |  | 0.44 |
| Yes | 78 (41.3) | 67 (35.4) | 77 (40.7) |  |
| No | 111 (58.7) | 122 (64.6) | 112 (59.3) |  |
| Alcohol intake |  |  |  | 0.539 |
| Yes | 19 (10.1) | 23 (12.2) | 26 (13.8) |  |
| No | 170 (89.9) | 166 (87.8) | 163 (86.2) |  |
| Family history of cancer |  |  |  | 0.785 |
| Yes | 42 (22.2) | 47 (24.9) | 47 (24.9) |  |
| No | 147 (77.8) | 142 (75.1) | 142 (75.1) |  |
| T category^a^ |  |  |  | 0.203 |
| T1 | 11 (5.8) | 13 (6.9) | 2 (1.1) |  |
| T2 | 16 (8.5) | 18 (9.5) | 17 (9.0) |  |
| T3 | 97 (51.3) | 94 (49.7) | 101 (53.4) |  |
| T4 | 65 (34.4) | 64 (33.9) | 69 (36.5) |  |
| N category^a^ |  |  |  | 0.356 |
| N1 | 96 (50.8) | 89 (47.1) | 109 (57.6) |  |
| N2 | 49 (25.9) | 53 (28.0) | 43 (22.8) |  |
| N3 | 44 (23.3) | 47 (24.9) | 37 (19.6) |  |
| Overall stage^a^ |  |  |  | 0.993 |
| III | 90 (47.6) | 91 (48.1) | 90 (47.6) |  |
| IVA | 99 (52.4) | 98 (51.9) | 99 (52.4) |  |
| IC cycle |  |  |  | < 0.0001 |
| 2 | 51 (27.0) | 146 (77.2) | 134 (70.9) |  |
| 3 | 119 (63.0) | 31 (16.4) | 45 (23.8) |  |
| 4 | 19 (10.0) | 12 (6.4) | 10 (5.3) |  |
| CCD (mg/m^2^) |  |  |  | 0.001^b^ |
| Median (range) | 160 (60-300) | 160 (30-300) | 160 (30-300) |  |
| ≥ 200 | 42 (22.2) | 18 (9.5) | 43 (22.8) |  |
| < 200 | 147 (77.8) | 171 (90.5) | 146 (77.2) |  |

Abbreviations: IC, induction chemotherapy; TPF, docetaxel plus cisplatin and 5-fluorouracil; PF, cisplatin plus 5-fluorouracil; TP, docetaxel plus cisplatin. CCD, cumulative cisplatin dose during radiotherapy.

^a^According to the 8^th^ edition of AJCC/UICC manual.

^b^*P* values were calculated by one-way ANOVA.

Supplementary Table S5. Results of multivariate analysis in the 189 pairs.

| Endpoint | Factor | Hazard ratio (95% CI) | *P* value |
| --- | --- | --- | --- |
| OS | Alcohol intake (Yes vs. No) | 1.618 (1.101-2.377) | 0.014 |
|  | N category (N2-3 vs. N0-1) | 1.696 (1.234-2.331) | 0.001 |
|  | Overall stage (IVA vs. III) | 1.877 (1.356-2.596) | < 0.0001 |
|  | Treatment (TPF plus CRT vs. PF plus CRT) | 0.580 (0.395-0.852) | 0.005 |
|  | Treatment (TP plus CRT vs. PF plus CRT) | 0.712 (0.503-1.008) | 0.056 |
| DFS | Alcohol intake (Yes vs. No) | 1.896 (1.327-2.710) | < 0.0001 |
|  | T category (T3-4 vs. T1-2) | 1.662 (1.066-2.592) | 0.025 |
|  | N category (N2-3 vs. N0-1) | 1.921 (1.417-2.605) | < 0.0001 |
|  | Overall stage (IVA vs. III) | 1.886 (1.389-2.562) | < 0.0001 |
|  | Treatment (TPF plus CRT vs. PF plus CRT) | 0.701 (0.484-1.016) | 0.061 |
|  | Treatment (TP plus CRT vs. TPF plus CRT) | 0.787 (0.562-1.103) | 0.164 |
| DMFS | Gender (Female vs. male) | 0.528 (0.316-0.880) | 0.014 |
|  | N category (N2-3 vs. N0-1) | 2.329 (1.580-3.432) | < 0.0001 |
|  | Overall stage (IVA vs. III) | 2.220 (1.492-3.303) | < 0.0001 |
|  | Treatment (TPF plus CRT vs. PF plus CRT) | 0.689 (0.425-1.115) | 0.129 |
|  | Treatment (TP plus CRT vs. PF plus CRT) | 0.866 (0.566-1.326) | 0.507 |
| LFFS | Overall stage (IVA vs. III) | 1.864 (1.152-3.017) | 0.011 |
|  | Treatment (TPF plus CRT vs. PF plus CRT) | 1.034 (0.625-1.711) | 0.897 |
|  | Treatment (TP plus CRT vs. PF plus CRT) | 0.567 (0.315-1.018) | 0.057 |

Abbreviations: OS, overall survival; DFS, disease-free survival; DMFS, distant metastasis-free survival; LFFS, locoregional failure-free survival; CI, confidence interval; IC, induction chemotherapy; CRT, concurrent chemoradiotherapy; Pre-DNA, pre-treatment plasma EBV DNA.

*P*-values were calculated using an adjusted Cox proportional hazards model with backward elimination and the following variables were included: gender (female vs. male), age (≥ 50y vs. < 50y), smoking (yes vs. no), drinking (yes vs. no), family history of cancer (yes vs. no), T category (T3-4 vs. T1-2), N category (N2-3 vs. N0-1), overall stage (IVA vs. III), cumulative cisplatin dose during radiotherapy (≥ 200 vs. < 200 mg/m^2^), induction chemotherapy cycle (2 vs. 3-4) and treatment groups (TPF plus CRT vs. PF plus CRT, TP plus CRT vs. PF plus CRT).
